# Combination of Mn-Mo Oxide Nanoparticles on Carbon Nanotubes through Nitrogen Doping to Catalyze Oxygen Reduction

**DOI:** 10.3390/molecules28145544

**Published:** 2023-07-20

**Authors:** Min Wang, Shilin Zhang, Juejin Teng, Shunsheng Zhao, Zhongtao Li, Mingbo Wu

**Affiliations:** 1 State Key Laboratory of Heavy Oil Processing, School of Chemical Engineering, China University of Petroleum (East China), Qingdao 266580, China; minwang@upc.edu.cn (M.W.); s22030041@s.upc.edu.cn (S.Z.); tengjuejin@foxmail.com (J.T.); s21030059@s.upc.edu.cn (S.Z.); 2 College of New Energy, China University of Petroleum (East China), Qingdao 266580, China

**Keywords:** MnMoO_4_/NCNT composites, nitrogen doping, oxygen reduction reaction, platinum group metal (PGM)-free catalyst, electrocatalysis

## Abstract

An efficient and low-cost oxygen catalyst for the oxygen reduction reaction (ORR) was developed by in situ growth of Mn-Mo oxide nanoparticles on nitrogen-doped carbon nanotubes (NCNTs). Doped nitrogen effectively increases the electron conductivity of the MnMoO_4_@NCNT complex and the binding energy between the Mn-Mo oxide nanoparticles and carbon nanotubes (CNTs), leading to fast charge transfer and more catalytically active sites. Combining Mn and Mo with NCNTs improves the catalytic activity and promotes both electron and mass transfers, greatly enhancing the catalytic ability for ORR. As a result, MnMoO_4_@NCNT exhibited a comparable half-wave potential to commercial Pt/C and superior durability, demonstrating great potential for application in renewable energy conversion systems.

## 1. Introduction

Fuel cells have gained increasing interest as a clean and efficient source of energy due to their high electrical energy conversion efficiency and environmental friendliness [1,2,3]. In many devices, the oxygen reduction reaction (ORR) is a vital process in the oxygen electrode [4,5,6]. Although platinum-based catalysts are considered the best ORR catalysts [7], they have limited reserves, high cost, and are intolerant to methanol. Moreover, these Pt electrocatalysts have poor long-term stability due to the migration and coalescence of Pt nanoparticles [8,9], which hinders their large-scale commercialization. Therefore, it is essential to explore new electrocatalysts with a reasonable price and excellent performance for ORR in alkaline media. Alternative electrocatalysts, including metal-free carbon material catalysts and transition metal-based catalysts, have been investigated [10,11,12]. Among the studied transition metals, Fe or Co with N-doped carbon form the M-N-C catalysts, which are considered as the most promising PGM-free catalysts. They exhibit encouraging ORR activity and stability even in harsh acidic media [13]. However, the inferior durability of Fe-N-C catalysts in membrane electrode assemblies (MEAs) is still a substantial technical barrier for fuel cell transportation applications. Even worse, hydrogen peroxide produced during the ORR will react with Fe^3+^/Fe^2+^ to form harmful hydroxyl and hydroperoxyl radicals by Fenton and attack organic ionomers, membranes, and catalysts. Although Co-N-C catalysts can alleviate the Fenton reactions to a certain degree, their relatively low intrinsic activity and high cost remain grand challenges. Mn-N-C catalysts, which are low-cost and have negligible activity toward the Fenton reactions, are highly desirable PGM-free catalysts [14]. Although single-atom catalysts have certain performance for ORR, according to the latest research, due to the improvement in the structural stability of the active center and the modulation of the electron cloud, bimetallic particles show higher activity and stability when compared with monometallic atomic particles [15]. To date, various nanocomposites with transition metals (Co, Fe, etc.) and heteroatom-doped carbons have demonstrated promising catalytic performance for the ORR.

Multi-transition metal oxides (MTMOs) are gaining attention as promising electrocatalysts due to their lower cost, higher electronic conductivity, and better electrochemical performance compared to single metal oxides [16]. MTMOs, such as FexMn_3_-xO_4_ and CoxNi_2_-xO_3_, are combinations of two or more transition metal oxides. Mn, in particular, is an intriguing series in spinel due to its earth abundance, low price, minimum environmental impact, and multiple valences, which exhibit high intrinsic activity for either the oxygen evolution reaction OER [17] or the ORR [18,19,20]. On the other hand, Mo, as an elementary ferromagnetic transition metal, is known for its outstanding electrical and magnetic characteristics, making it beneficial for electrochemical energy storage [21,22,23]. Therefore, integrating MnMo alloys or their oxides as a bi-metal electrocatalyst could provide additional synergistic properties during electrocatalysis. The dissimilarity in lattice strain between Mn and Mo would create more active defects and varied redox potentials, improving the electrochemical performance of the catalyst.

The combination of transition metal oxide particles with conductive matrices, such as graphene, CNTs, or carbon fibers, can improve their electrochemical performance [24]. Some N-containing precursors are adopted during the combination process for the following reasons: (1) they act as surfactants to enhance the compatibility and interaction between the carbon support and inorganic particles due to their high polarity, and (2) they improve conductivity and catalytic activities for the ORR due to increased active sites during N-doping [25,26]. To optimize ORR catalysts, suitable nitrogen/transition-metal precursors and carbon supports should be precisely selected and controlled. In particular, nitrogen-doped CNTs have received increasing attention as ORR catalysts in the past decade due to their excellent conductivity and strong mechanical/chemical stability [27,28].

In this paper, we present a facile approach for synthesizing MnMoO_4_@NCNT composites to catalyze the ORR. Firstly, CNTs were acid-treated and then coated with transition metal (Mn, Mo) nanoparticles through a solvothermal process. Dicyanogen was adopted as a “junctor” during the coating process, which resulted in smaller and more uniform Mn-Mo nanoparticles being co-deposited with metal oxide on the CNTs. The resulting MnMoO_4_@NCNT composites, after calcination at 500 °C, possessed highly active catalytic sites, an interpenetrated hierarchical conductive matrix, and a large specific surface area, leading to enhanced ORR activity.

## 2. Results and Discussion

**Figure 1:**
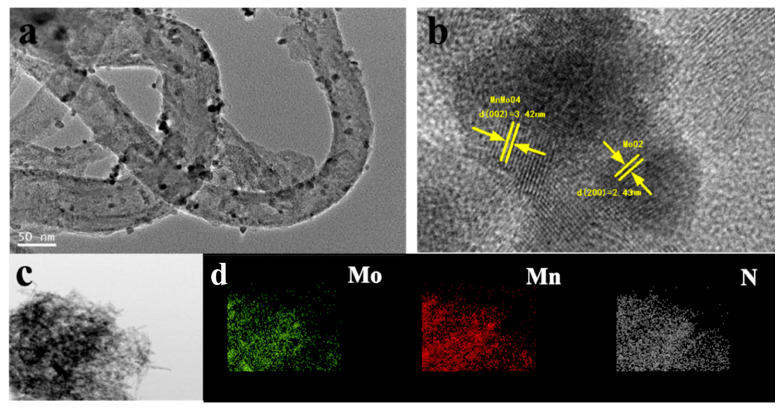
TEM images of (**a**,**b**) MnMoO_4_@NCNT pyrolyze at 500 °C; (**c**,**d**) Color EDS mapping images of a single MnMoO_4_@NCNT nanohybrids.

To provide further evidence of the structure of MnMoO_4_@NCNT, high-resolution transmission electron microscopy (HRTEM) images were obtained. As depicted in Figure 1a, the nanocrystals of Mn-Mo were well dispersed on the surface of CNTs, with an average size of approximately 10 nm. No obvious congestion of nanoparticles was observed in MnMoO_4_@NCNT nanohybrids, indicating that the binding energy between Mn-Mo and pre-treated CNTs was stronger than that between MnMoO_4_ particles themselves. In Figure 1b, the HRTEM image of MnMoO_4_@NCNT nanohybrids clearly showed the lattice fringes of MnMoO_4_, with an interplanar spacing of 0.25 nm corresponding to the d-spacing of the (111) plane of tetragonal MnMoO_4_ crystals. The thickness of CNTs in the MnMoO_4_@NCNT catalyst was estimated to be approximately 3.0~4.0 nm. The EDS (Energy Dispersive Spectroscopy) elemental mapping of a single MnMoO_4_@NCNT nanohybrid was presented in Figure 1d, confirming the uniform distribution of Mn and Mo elements in the MnMoO_4_ nanoparticles. Moreover, the N atoms were consistently dispersed on the surface of CNTs, even in domains that bound with MnMoO_4_ nanocrystals. This indicated that the N source possessed higher reactivity or stronger interaction with pre-treated CNTs and Mn-Mo nanocrystals. The small size of MnMoO_4_ oxide nanoparticles combined with their dispersion in CNTs facilitates fast electron transport between the carbon matrix and MnMoO_4_ oxide nanoparticles, leading to efficient electrical conductivity and enhancing the ORR activity.

**Figure 2:**
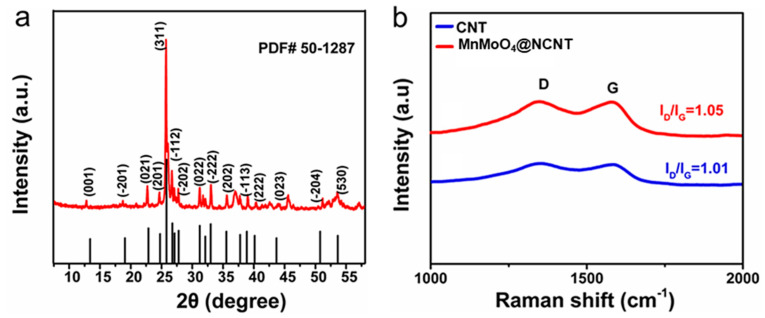
(**a**) XRD patterns of MnMoO_4_@NCNT; (**b**) Raman spectra of CNTs and MnMoO_4_@NCNT.

Figure 2a displays the powder X-ray diffraction (XRD) pattern of the MnMoO_4_@NCNT catalyst. The sharp diffraction peak at 2θ = 26.3° corresponds to the (111) plane of graphite structure, which is consistent with the crystalline planes of carbon in Joint Committee on Powder Diffraction Standards (JCPDS) card 75-1621. The well-defined peaks observed around 2θ = 13.1°, 22.7°, 25.7°, 31.2°, 32.1°, 35.6°, 40.3°, 43.9°, and 54.1° match well with JCPDS card 50-1287, indicating the presence of Manganese molybdate on the MnMoO_4_@NCNT. Furthermore, Raman spectra (Figure 2b) were used to examine the degree of disorder in the carbon material. The D band located at 1350 cm^−1^ and G band near 1588 cm^−1^ are related to the disordered and ordered structure of the carbon material, respectively. The peak area ratio of a (*I_D_/I_G_*) was 1.05, and b (*I_D_/I_G_*) was 1.01, indicating a higher degree of disorder. This finding correlates highly with the doping of N and presence of defects on CNTs. However, the presence of MnMoO_4_ leads to a higher degree of graphitization, which is consistent with the XRD results.

**Figure 3:**
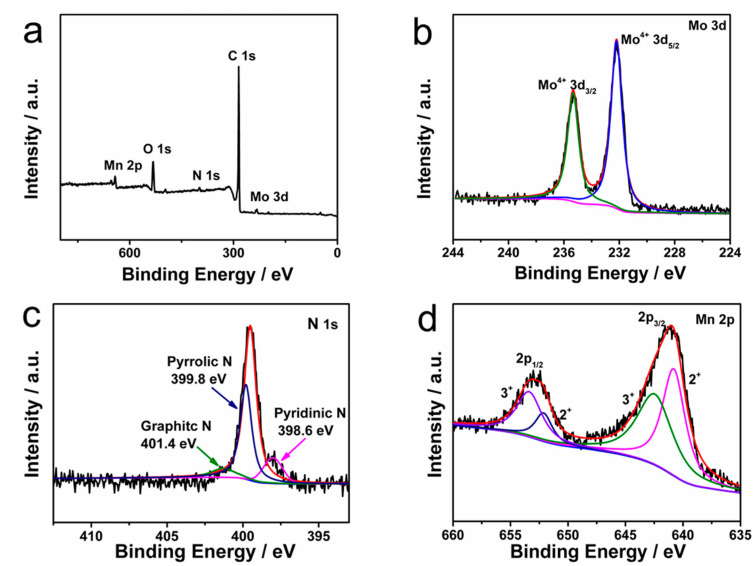
(**a**) The full XPS spectrum of MnMoO_4_@NCNT; (**b**) Mo 3d; (**c**) N 1s; and (**d**) Mn 2p XPS spectra for MnMoO_4_@NCNT.

X-ray photoelectron spectroscopy (XPS) was used to determine the chemical compositions of MnMoO_4_@NCNT. The wide scan spectra of the three samples showed photoelectron lines at binding energies of 285, 400, 645, and 232 eV, which corresponded to C 1s, N 1s, O 1s, Mn 2p, and Mo 3d, respectively (Figure 3a) [29]. The N 1s spectrum was deconvoluted into three components centered at 398.6 eV (pyridinic N), 399.8 eV (pyrrolic N), and 401.4 eV (graphitic N), which indicated the presence of the nucleophile substitution of dicyandiamide and pre-treated CNT matrix [30]. The N content of MnMoO_4_@NCNT nanohybrids was calculated to be ~2.0 at%. The high N-containing rate could increase conductivity and afford more electrochemically active sites during the oxygen reduction reaction. Additionally, the N-containing groups could enhance the binding energy between Mn-Mo and CNTs, acting as a “bridge”, and thus be beneficial for enhancing electrochemical performances of materials. The Mn 2p spectrum featured two main peaks of Mn 2p_3/2_ and Mn 2p_1/2_ entered at 642.5 eV and 652.5 eV, respectively, with a spin energy separation of 10.5 eV that was in good agreement with reported data of Mn 2p_3/2_ and Mn 2p_1/2_ in MnMoO_4_. After refined fitting, the spectrum could be deconvolved into four peaks at binding energies of 642.5 eV (Mn^2+^), 643.9 eV (Mn^3+^), 653.1 eV (Mn^2+^), and 653.7 eV (Mn^3+^). The Mo 3d XPS spectra of MnMoO_4_@NCNT nanohybrids exhibited two characteristic peaks (232.0 eV and 235.0 eV), corresponding to the Mo 3d_3/2_ and Mo 3d_5/2_ spin-orbit peaks of Mo^4+^. It was reasonable to conclude that the solid-state redox couples of MnMoO_4_ in MnMoO_4_@NCNT samples exhibited higher electrical conductivity and electrochemical activity due to the synergetic effects of multiple valences of the cations [31].

**Figure 4:**
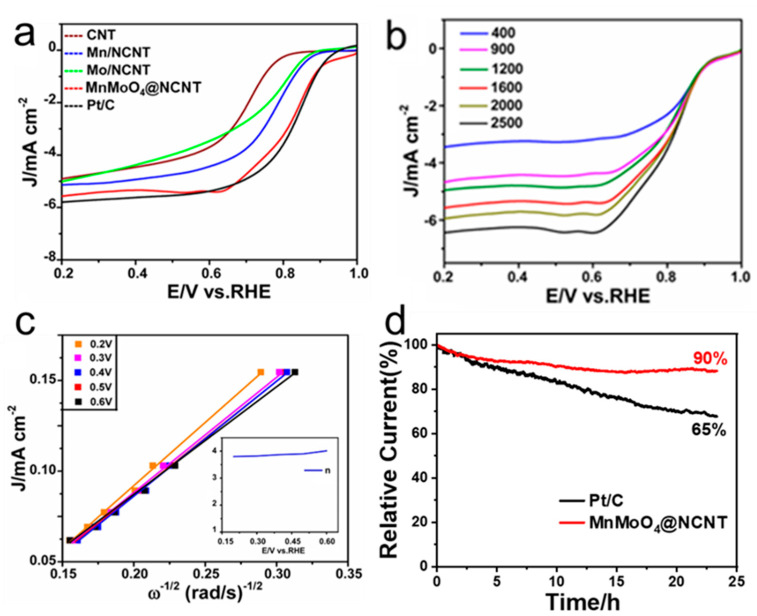
(**a**) LSV curves of above catalysts in O_2_-saturated 0.1 mol L^−1^ KOH at a scan rate of 10 mV s^−1^, rotating speed 1600 rpm; (**b**) LSV curves of MnMoO_4_@NCNT in O_2_-saturated 0.1 mol L^−1^ KOH with different rotating speed at a scan rate of 10 mV s^−1^; (**c**) the K-L plot and the electron transfer number of MnMoO_4_@NCNT; (**d**) durability test of MnMoO_4_@NCNT and Pt/C in O_2_-saturated 0.1 mol L^−1^ KOH.

To gain insight into the electrocatalytic ORR activities of the prepared samples, linear sweep voltammetry (LSV) measurements were performed in O_2_-saturated 0.1 M KOH solution. As shown in Figure 4a, all the onset potentials of MnMoO_4_@CNT were comparable to that of Pt/C, and MnMoO_4_@NCNT outperformed other samples in terms of disk current density and half-wave potential (E_1/2_). At 1600 rpm, E_1/2_ of MnMoO_4_@NCNT was 0.83 V, close to that of the Pt/C catalyst. Moreover, MnMoO_4_@NCNT had the highest current density of 5.6 mA cm^−2^ at 1600 rpm, while Mn@NCNT, Mo@NCNT, and Pt/C had current densities of 5.1, 5.0, and 5.9 mA cm^−2^ with the same loading and rotating speed, respectively. The mixed oxidation states of Mn^2+^ and Mo^4+^ increased the electronic conductivity of 3D conductive networks and more electrochemically active surface sites [32,33,34,35], leading to the formation of M-OH or M-OOH species on the catalytic surface to absorb more oxygen [36]. Therefore, the ORR activity of MnMoO_4_@NCNT was better than that of other catalysts (Mn/NCNT, Mo/NCNT). The combination of MnMoO_4_ with N-doped CNTs could achieve high catalytic activity due to the increased conductivity through the reduction during N-doping at high temperature, the accelerated charge and mass transfer capability, and more catalytic active sites resulting from the combination of MnMoO_4_ nanoparticle nanocrystals on CNTs. To investigate the primary electron reduction pathway of different catalysts, we conducted LSV measurements at various rotating speeds as shown in Figure 4b. Figure 4c illustrates the K-L plot of MnMoO_4_@NCNT. The in situ-grown MnMoO_4_ on CNTs, bound by carbon nitride, provides continuous and multiple pathways for electron transfer. Moreover, the voids among the nanocomposites ensure smooth mass transfer to active sites, improving the ORR diffusion kinetics. The electron transfer number for MnMoO_4_@NCNT was approximately 4.0, as shown in the Figure 4c inset, indicating an exclusive four-electron pathway, consistent with the ORR catalyzed by a Pt/C catalyst.

The stability of MnMoO_4_@NCNT was evaluated in O_2_-saturated 0.1 M KOH solution through chronoamperometry. Results in Figure 4d suggest that MnMoO_4_@NCNT may be a viable substitute for commercial Pt/C. After continuous ORR testing at 0.5 V (vs. RHE) for about 24 h, the current of the commercial Pt/C electrode exhibited a 35% loss. In contrast, the current of MnMoO_4_@NCNT decreased only about 10%. This improved stability can be attributed to the unique structure of the nanohybrid, in which polymerized dicyandiamide effectively “bridges” the inorganic particles and CNTs together, enhancing the catalyst’s stability. Comparison of the ORR performance of different PGM-free catalysts is shown in Table 1.

## 3. Experimental Section

### 3.1. Reagents and Chemicals

Nafion (5 wt%) MWCNTs were obtained from Sigma-Aldrich. Pt/C (20 wt%) was obtained from Johnson-Matthey. Ultrapure water (Millipore, 18.4 MΩ cm^−1^) was used throughout the whole experiment. All other chemicals in the experiment were of analytical grade, purchased from Aladdin Industrial Corporation (Shanghai, China), and used as received without further purification.

### 3.2. Preparation of MnMoO_4_@NCNT Composites

**Figure 5:**
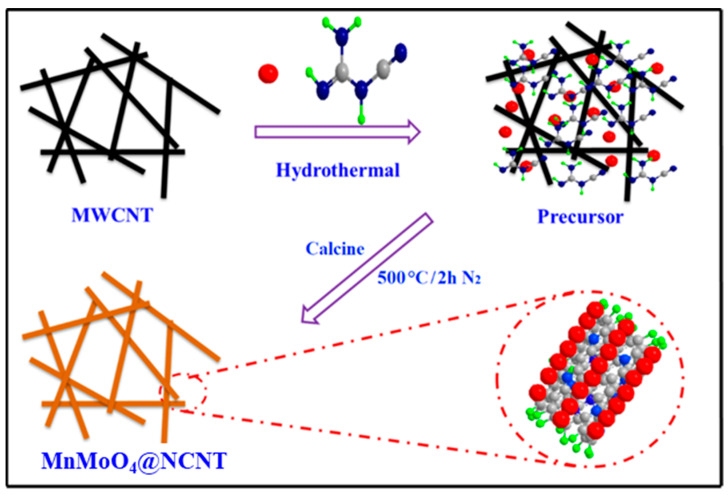
Schematic illustration of the preparation process for the MnMoO_4_@NCNT catalyst, and the possible microstructure in MnMoO_4_@NCNT catalyst.

The synthesis of MnMoO_4_@NCNT composites is illustrated in Figure 5, using a straightforward hydrothermal method. First, H_8_MoN_2_O_4_ (196.0 mg), dicyandiamide (250 mg), potassium permanganate (158.03 mg), and ultrapure water (40 mL) were stirred at room temperature. The resulting mixture was then transferred and sealed into an 80 mL Teflon-lined stainless-steel autoclave, which was heated to 120 °C for 4 h and subsequently allowed to cool to room temperature. The black product was then washed with deionized water and ethanol five times each, and the resulting mixture was dried via freeze drying for 10 h. The dried precursor was placed into a crucible and heated to 500 °C at a rate of 2.5 °C/min for 2 h in a nitrogen atmosphere. Once cooled to room temperature, the MnMoO_4_@NCNT was obtained. Mn@CNT and Mo@CNT were prepared using a similar procedure, without the addition of H_8_MoN_2_O_4_ and dicyandiamide, respectively.

### 3.3. Materials Characterization

The synthesized samples were characterized using various analytical techniques. XRD was carried out using a X’Pert PRO MPD instrument from Holland with Cu Ka radiation. Transmission electron microscopy (TEM) and field emission scanning electron microscopy (FE-SEM) were used to investigate the structure and morphology of the samples, using a JEM-2100UHR and S4800 microscope from Japan, respectively. The crystallinity of the samples was determined by Raman analysis using a Jobin-Yvon Labram-010 Raman spectrometer. XPS results were obtained using an ESCALab MKII instrument.

### 3.4. Electrochemical Measurement

Electrochemical experiments were conducted at room temperature in a three-electrode cell connected to a CHI-760E electrochemical analyzer. A platinum plate served as the counter electrode and Ag/AgCl as the reference electrode. The ORR tests were performed in O_2_-saturated 0.1 M KOH solution at room temperature. In rotating disk electrode (RDE) measurements, the working electrode was a 4 mm diameter glassy carbon (GC) disk. For each sample, including the commercial 20% Pt/C, 2.0 mg of the sample was dispersed in an 800.0 μL ethanol solution with 5.0 μL of Nafion solution and sonicated for 30 min. A total of 10.0 μL of well-dispersed catalyst suspension was dropped onto the glassy carbon electrode surface (4.0 mm in diameter, Pine Research Instrumentation) and allowed to dry at room temperature for 2.0 min (catalyst loading: ~0.20 mg cm^−2^).

To investigate the electrocatalytic performance of the as-prepared catalysts, LSV was performed in 0.1 M KOH solution. Prior to each test, the KOH solution was saturated with ultrahigh pure O_2_ for 30 min. All potentials reported herein were calibrated with respect to the reversible hydrogen electrode (RHE) using the equation E_RHE_ = E_Ag/AgCl_ + 0.0591pH + E^θ^_Ag/AgCl_. LSV was tested to evaluate the electron transfer number per O_2_ for the ORR, at a rotation rate ranging from 400 to 2500 rpm. The electron transfer number for ORR can be calculated through Koutecky–Levich plots at different electrode potentials. Rotating ring disk electrode (RRDE) measurements were used to monitor the yield of peroxide species (HO_2_^−^) and evaluate the electron transfer number per oxygen molecule during the process of the ORR [42]. For comparison, commercial Pt/C (20 wt %), Mn@NCNT, and Mo@NCNT were also tested using the same procedure.

## 4. Conclusions

In summary, we have developed an efficient and low-cost oxygen catalyst for ORR by the in situ growth of MnMoO_4_ oxide nanoparticles on nitrogen-doped carbon nanotubes (N-CNTs). The introduction of nitrogen brought about several advantages, including increased conductivity of the complex through high-temperature reduction and assistance in the combination of MnMoO_4_ oxide nanoparticles with CNTs. This led to an accelerated charge and mass transfer capability and more catalytically active sites. The resulting product showed greatly improved catalytic ability for ORR, with a half-wave potential of 0.83 V (vs. RHE), which is close to that of Pt/C catalysts. Doping nitrogen into the nanoparticles improved catalytic activity and promoted both electron and mass transfer during ORR. MnMoO_4_@NCNT exhibited a current density of 5.6 mA cm^−2^ and the 4e^−^ electron transfer process, making it a promising candidate for developing renewable energy conversion systems.

## Figures and Tables

**Table 1:** Comparison of the ORR performance.

Electrocatalyst	Electrolyte	Onset Potential (V vs. RHE)	Half-Wave Potential (V vs. RHE)	Electron Transfer Number	Ref.
MnMoO_4_@NCNT	0.1 M KOH	0.9	0.83	4	This work
Mn-N-C	0.5 M H_2_SO_4_	0.9	0.69	4	[14]
Fe-Mn/N-C	0.1M HClO_4_	0.95	0.80	2	[15]
Mn-N-C	0.5 M H_2_SO_4_	0.9	0.8	4	[13]
Co-N-C	0.1 M H_2_SO_4_	0.82	0.78	4	[37]
Fe-N-C@MXene	0.1 M KOH	0.9	0.887	4	[38]
N-Fe_2_MoC-GC	0.1 M KOH	0.95	0.887	4	[39]
FeCo/NC-Mo_2_TiC_2_	0.1 M KOH	1.017	0.887	2	[40]
Fe-Mn/NC	0.1 M KOH	1.015	0.904	4	[41]

## Data Availability

The data that support the findings of this study are available from the corresponding author upon reasonable request.
